# The effect of caregiving on bereavement outcome: study protocol for a longitudinal, prospective study

**DOI:** 10.1186/s12904-015-0009-z

**Published:** 2015-03-15

**Authors:** Lauren J Breen, Samar M Aoun, Moira O’Connor

**Affiliations:** School of Psychology & Speech Pathology, Faculty of Health Sciences, Curtin University, GPO Box U1987, Perth, Western Australia 6845 Australia; School of Nursing and Midwifery, Faculty of Health Sciences, Curtin University, Perth, Australia

**Keywords:** Bereavement, Family caregivers, Palliative care, Longitudinal

## Abstract

**Background:**

The aim of the current study is to determine the effects of caregiving on bereavement outcome. The study will address two important gaps in the research literature: (1) the relationship between pre-death distress and post-death outcomes and (2) family caregivers’ anticipation and preparation of the death of the person for whom they care.

**Methods/Design:**

We will conduct a longitudinal, prospective study of adult family caregivers of adult patients receiving palliative care. All participants will complete a questionnaire administered at four points – approximately 4–8 weeks prior to bereavement, and 3–4, 6–7, and 9–10 months post-bereavement. The questionnaire includes measures of multidimensional caregiving experiences (strain, distress, positive appraisals, and family wellbeing), caregiver prolonged grief, multidimensional grief responses (despair, panic behaviour, blame and anger, detachment, disorganisation, and personal growth), prolonged grief, quality of life, general health (psychological and physical) and demographics. These caregivers’ data will be compared to a comparison group matched for age, sex, and postcode, allowing the caregivers’ general health and quality of life to be compared to a normative group. The caregivers will also be invited to participate in a semi-structured interview about preparing for impending bereavement.

**Discussion:**

This is the first study to address the methodological limitations in the current literature and will likely make a significant contribution to both our understanding of caregiving on bereavement outcome and to bereavement care offered in palliative and hospice settings.

## Background

In Australia, as in many countries, a typical death involves a considerable period of time where family members, usually spouses or adult offspring, care for people with chronic and disabling conditions in the home [1]; this caregiving role continues when, or if, the patient is admitted to an in-patient palliative care or hospice service near the end-of-life [2,3]. While much is known about the experiences and needs of family caregivers prior to the death [4,5], less is known about the effect of caregiving on bereavement outcome [6,7], and the literature that is available is compromised by three methodological limitations.

First, the relationship between caregiving and bereavement outcome is typically investigated using cross-sectional and/or retrospective designs [8-11] over a very short period of time such as 80 days [12], or with data collected at only two time periods [10,12,13]. A longitudinal, prospective approach with several data collection points is necessary to assess changes in the grief response over time. One prospective, longitudinal study of 217 informal caregivers of people recently placed in nursing homes used four data collection points over 18 months and demonstrated that preparing for death eases post-bereavement adjustment for caregivers [14].

Second, these studies do not typically include adequate comparison groups [14-16]. The omission of comparison groups, matched according to age, sex, and income, overlooks the potential that high distress may be a likely consequence of caring for a dying loved one. Without appropriate comparison, we cannot determine if a reduction in caregivers’ distress over time indicates a return to ‘normal’ functioning [6]. Non-caregivers provide an ideal control group because the caregivers’ distress in relation to non-caregivers’ (a normative group) can be assessed.

Third, caregivers’ experiences of anticipating, expecting, and preparing for the death of the person for whom they care tend to be overlooked [7]. However, research shows that, even for caregivers who perform high-intensity care over months and years, approximately a quarter of caregivers report being unprepared for the death [17,18]. Caregivers do not tend to describe themselves as prepared for the death of the person for whom they care [19] and they are often hesitant to confront the impending death of their family member [20]. Clearly, there is a need for research on caregivers’ perspectives on what it means to anticipate, expect, or prepare for the death of the person for whom they care.

### The current study

In this study we will address the three methodological limitations in the current literature by taking a longitudinal, prospective approach with a comparison group, which will be complemented by semi-structured interviews. The following research questions are proposed:

1. Does family caregivers’ pre-death (anticipatory) grief and distress predict post-death adjustment?
2. How does the former caregivers’ post-bereavement distress compare with that of non-caregivers?
3. What are caregivers’ experiences and understandings of anticipating, expecting, and preparing for the death of the person for whom they are caring?

## Methods

### Study design

The mixed-methods study has two components: First, a longitudinal study using quantitative measures of primary caregivers’ and matched comparisons’ general health, emotional distress, and quality of life pre-and post-bereavement, in order to determine the changes over time, and how the recently bereaved caregivers’ distress compares with the matched comparisons. This comparison group (people who are not currently caring for a person with life-limiting illness, or have not occupied this role in at least the past two years) provides an ideal way to assess the extent to which caregivers return to ‘normal’ levels on these measures post-bereavement.

The second component of the study involves semi-structured interviews with a subset of the primary caregivers to explore their understandings and experiences of anticipating and preparing for the death of the person for whom they are caring. The interviews will be informed by grounded theory, which is a systematic and data-driven methodology aimed at uncovering the phenomenological character of, and generating theory about, a phenomenon [21]. In this case, the phenomenon is caregivers’ understandings and experiences of anticipating and preparing for the death of the person for whom they care.

### Participants and recruitment

Participants will be recruited from several palliative care services (in-patient, consultative, and community-based) in Western Australia. Inclusion criteria include: 1) aged 18 years and over, 2) are an informal caregiver of a patient receiving palliative care, and 3) the patient’s health is considered by the treating healthcare professionals to be ‘stable’. Participants will be excluded if they: 1) exhibit cognitive problems, as determined by the healthcare team, and/or 2) do not speak or understand English.

We aim to recruit 82 participants (41 caregivers and 41 matched comparisons). The matched comparisons will comprise adults matched for age, sex, and postcode (as a proxy for income). The comparisons will be recruited via advertisements on the university’s radio station, advertisements in print media [22], and flyers placed on community noticeboards. Potential comparisons will be excluded if they: 1) currently care for anyone with a life-limiting or terminal illness and/or 2) were bereaved in the previous two years.

The primary caregivers will be recruited from palliative care services in collaboration with healthcare staff. In Australia, patients tend to be referred to palliative care and hospice facilities when death is imminent; usually when their doctor estimates that death will occur within two months [23]. An examination of the admission data will provide a relatively rigorous yet parsimonious method of identifying caregivers approximately 4–8 weeks before the death of the person for whom they care.

### Data collection

Self-report measures will be administered at four points – approximately 4–8 weeks prior to bereavement (baseline), and 3–4 (follow-up time 1), 6–7 (follow-up time 2), and 9–10 months (follow-up time 3) post-bereavement. The collection of these data for caregivers and matched comparisons will address the first and second research questions.

At the first data collection point (baseline), the caregivers will be invited to participate in a semi-structured interview to explore their perspectives on anticipating and preparing for the death of the person for whom they care. It is anticipated that approximately 20 participants will be interviewed, depending on data saturation, which is achieved when themes are recurring and no new information is emerging. Interviews will occur at each participant’s home, the palliative care service site, or at a mutually-convenient venue. These interviews will address the third research question.

### Ethical issues

Caregivers and the recently-bereaved may be vulnerable yet they usually characterise participation in research as helpful or not harmful [24-26]. Given the ethical obligations to assist research participants identified as distressed [27], all former caregivers returning self-reports indicative of symptomatology will be referred for psychological services immediately. Information about appropriate services will also be included in the participant information document. The study has been approved by the South Metropolitan Health Service Human Research Ethics Committee (Ref number: 12/284), Hollywood Private Hospital (Ref number: HPH378], and Curtin University Human Research Ethics Committee (Ref number: HR131/2012).

### Measures

Caregivers will be asked to complete a set of measures to obtain prospective, longitudinal data on their grief responses, distress levels, and adjustment over time. In addition to measuring the grief response, the measures will provide data on symptoms of psychiatric disorders, and beneficial adaptations to loss (e.g., growth).

Family appraisal of caregiving questionnaire for palliative care (FACQ-PC) – a 25-item measure of the multidimensional caregiving experiences and comprises four scales – strain, distress, positive appraisals, and family wellbeing. The measure and its scales have high internal consistency reliability (0.73-0.86) and acceptable construct and convergent validity [28]. This measure will only be completed by the caregivers and only at the first data collection point.

Prolonged grief disorder (PG-12) caregiver version – a 12-item measure of complicated grief arising from the impending death of a loved one [29]. This measure has high internal consistency reliability [30] and will only be completed by the caregivers and only at the first data collection point.

Hogan grief reaction checklist – a 61-item measure of the multidimensional nature of grief. The authors report high internal consistency, test-retest reliability, and construct, convergent, and divergent validity [31] and predictive validity [32]. Importantly for the proposed study, the items do not require each participant to evaluate symptoms specifically in relation to a deceased loved one and, as such, it is appropriate for the pre- and post- measures of emotional state. The Checklist will be adapted slightly for use with the comparison group. Doing so will require altering items in the Despair factor; the five remaining factors (panic behaviour, blame and anger, detachment, disorganisation, and personal growth) will remain unaltered.

Short form health survey (SF-12v2) – The SF-12v2 is the standard 12-item measure of general health and wellbeing (psychological and physical). This scale is a reliable and valid measure of health status which provides a measure for health for clinical and economic appraisal [33] with questions concerning physical health problems, bodily pain, general health perceptions, vitality (energy/fatigue), social functioning, role limitations, and general mental health (psychological distress and psychological wellbeing). Reliability estimates range from 0.93 to 0.95. Norms for the Australian population are available for this questionnaire [34], allowing an additional comparison between the bereaved former caregivers and the ‘normal’ population.

The quality of life index – The QOL Index is a two-item quality of life instrument that has demonstrated construct validity and internal consistency [35]. This tool will provide a global quality of life rating, rather than a multi-dimensional detailed assessment. This type of assessment has been shown to result in less missing data compared with studies using longer, complex QOL instruments.

The prolonged grief measure (PG-13) – a 13-item measure to assess symptoms of Prolonged Grief Disorder. The scale is reliable and valid and maps to criteria proposed for diagnostic nosology [36]. This measure will only be given to the former caregivers (not the matched comparisons) and only at the third and fourth data collection points.

Demographic questionnaire – A short demographic questionnaire will be used at the first data collection point to collect information such as age, sex, family/household characteristics, household income, and time since diagnosis of illness, and will aid the matching of caregivers with comparisons. The caregivers’ questionnaire will also include questions on the variability of the type and ‘level’ of care provided (e.g., types of tasks and the time involved).

Semi-structured Interview – the caregivers who express interest in an interview will be asked a series of open-ended questions. The questions will cover topics such as the illness trajectory of their loved one, when caregiving began and its forms, the impact the caregiving role has had, whether and where the caregiver accesses and/or continues to access support to assist in the caregiving role, perceived benefits of the caregiver role, understandings and experiences of anticipation of death of the family member, the extent to which the caregiver feels prepared for the death of the loved one, when death is expected to occur, and whether and how the healthcare/service delivery for the patient and the family could be improved. It is expected that these interviews will be up to one hour in duration.

### Sample size calculation and analyses

Assuming medium effect sizes, 41 per group are required for adequate statistical power (G*Power) [37]. Previous longitudinal studies of caregivers and bereaved individuals (either retrospectively or without adequate comparison groups) report participant attrition rates around 15% [8,15,16]. Thus, assuming a participant withdrawal rate of 15% for both groups throughout the proposed study, the starting sample size is expected to be a minimum of 96 (48 caregivers and 48 comparisons) in order to retain statistical power.

A Generalised Linear Mixed Model (GLMM) will be used to assess the caregiver group’s adaptation/distress over time. Differences between the caregivers and comparisons on the Hogan Grief Reaction Checklist, SF-12v2, and QOL Index will be examined, with Bonferroni adjustments to alpha. The bereaved former caregivers’ PG-13 scores will be computed at 6–7 months and 9–10 months post-bereavement to determine the proportion meeting diagnostic criteria. The data will also be used to conduct a series of regression analyses:

1. A simple regression model to investigate the relationship between time and grief scores for the bereaved former caregivers;
2. A multiple regression analysis with time and pre-bereavement grief scores as predictor variables and each post-bereavement grief measures as criterion variables; and
3. A multiple regression analysis to determine variables that predict symptomatology for complicated grief at 6–7 months and 9–10 months (i.e., demographics, scores on the SF-12v2, Hogan Grief Reaction Checklist, and the QOL Index).

All interviews will be audio-recorded and transcribed verbatim to ensure authentic records for analysis. The transcripts will be uploaded into the NVivo10 program [38]. Interim analysis will begin as soon as possible after each interview to optimise interpretations of the data and to aid theoretical sampling of the participants. Analysis will be driven by the three primary techniques used concurrently in grounded theory – coding, memoing, and diagramming [21]. Coding involves abstracting from the data in order to begin developing categories, which are then linked to achieve conceptual order. Memo writing aids the exploration of commonalities and differences in the data, and provides hypotheses or questions, and reflections. Diagramming results in a visual representation of the relationships between codes and categories generated from the entire data pool and assists in the identification of relationships between concepts and categories. Data collection and analysis will occur concurrently until no new information is uncovered, as identified via the recurrence and verification of data and themes from the participants. Rigor for the study will be ensured through addressing the components of credibility, auditability, and fittingness [39].

## Discussion

This study is the first to address two important gaps in our knowledge of caregiving and bereavement: (1) the relationship between caregivers’ pre-death grief and distress and post-death outcomes, and (2) caregivers’ understandings and experiences of the anticipating and preparing for the death of the person for whom they care. This study offers significant advantages over previous efforts to determine the effects of caregiving on bereavement outcome. First, the longitudinal, prospective approach with four data collection points commencing during caregiving is necessary to assess changes in the grief response over time. Second, the comparison group permits the caregivers’ distress to be compared against non-caregivers’, in order to determine the presence of long-term distress over and above community levels. Third, these quantitative data will be complemented by semi-structured interviews that will be used to explore caregivers’ anticipation and preparation of the death of the person for whom they care. While the proposed sample size is small, the study is sufficiently powered. The study is therefore innovative and unique because it overcomes three methodological flaws within the one study and will therefore provide a novel contribution to our understandings of the relationship between caregiving and bereavement.

The study’s ability to inform policy and practice in the provision of services for caregivers following bereavement will be welcomed within palliative care. Palliative care and hospice services grapple with how best to use their limited resources to support bereaved former caregivers [40]. It is increasingly recognised that while most bereaved people do not require formal bereavement services, a considerable minority will benefit from non-specialised support (e.g., mutual-help groups, trained volunteer support), and an even smaller proportion will experience persistent psychiatric distress, such as Prolonged Grief Disorder, requiring specialist intervention [36,41,42]. However, palliative care services offer bereavement services with little regard to support need [40] and the necessity for “clear evidence to guide development and allocation of bereavement programs in palliative care” [43], p. 230 has been noted.

Additionally, the findings will inform services and supports offered to caregivers, pre- and post-bereavement. Health sectors face a huge financial strain in providing end-of-life care [44,45], yet much of the costs of caring are absorbed by family caregivers [46]. Outcomes of this project will significantly improve the care given to those who devote themselves to the care of very ill family members. Supporting caregivers is essential for their wellbeing as well as for the cost-effectiveness of end-of-life care.

## Abbreviations

SF-12v2: Short form health survey
QOL: Quality of life
PG-13: Prolonged Grief measure
